# Mutations in the *NKX2.1* and the *PAX8* genes in a boy with thyroid dysgenesis, respiratory and neurological disorders

**DOI:** 10.1186/2194-7791-2-S1-A9

**Published:** 2015-07-01

**Authors:** Pia Hermanns, Małgorzata Kumorowicz-Czoch, Joachim Pohlenz

**Affiliations:** Department of Pediatrics, Johannes Gutenberg University Medical School, Mainz, Germany; Department of Pediatric and Adolescent Endocrinology, Chair of Pediatrics, Polish-American Institute of Pediatrics, Jagiellonian University Medical College, Cracow, Poland

## Background

Brain-lung-thyroid syndrome (BLTS) is a rare disorder characterized by congenital hypothyroidism (CH), infant respiratory distress syndrome (IRDS) and benign hereditary chorea (BHC). BLTS is caused by mutations in the *NKX2.1* gene.

## Objective

We describe a patient with TD, respiratory disease and cerebral palsy and who has two heterozygous mutations in the *PAX8* (p.E234K) and the *NKX2.1* (p.A329GfsX108) genes. *In vitro* studies were performed to functionally characterize these mutations.

## Patient

The 12 years-old boy was diagnosed to have congenital hypothyroidism (CH) at neonatal screening, with a serum TSH of 49.5 mU/L (N: 0.4-9) and a T4 of 3.76 µg/dl (N: 50-197). The thyroglobulin value was 3.18 ng/ml, N<55 and hypoplastic thyroid in ultrasonography. After birth, he developed severe respiratory failure, seizures and an ischemic cerebral infarction. He was diagnosed with cerebral palsy and symptomatic epilepsy and developed a considerable psychomotor retardation. Currently he is euthyroid under L-thyroxine supplementation. His sister and the parents are healthy and euthyroid.

## Methods

We introduced the two identified mutations into expression vectors and transiently transfected them into HeLa cells. In EMSA studies we tested for the DNA binding capability of the two mutated transcription factors.

## Results

The PAX8 mutation was normally located to the nucleus and showed a normal transactivation of and normal binding to the known downstream targets. In contrast the NKX2.1 mutation did not show any transactivation ability due to the loss of the capability to bind to DNA. It remains to be elucidated whether the NKX2.1 mutation localizes to the nucleus.

## Conclusions

The *NKX2.1* mutation might be responsible for the phenotype observed in our patient. The synergistic effect is completely abolished by the NKX2.1 mutation when cells were co-transfected with the PAX8 expression constructs.

